# Protein Phosphatase, Mg^2+^/Mn^2+^-dependent 1A controls the innate antiviral and antibacterial response of macrophages during HIV-1 and *Mycobacterium tuberculosis* infection

**DOI:** 10.18632/oncotarget.8190

**Published:** 2016-03-18

**Authors:** Jim Sun, Kaitlyn Schaaf, Alexandra Duverger, Frank Wolschendorf, Alexander Speer, Frederic Wagner, Michael Niederweis, Olaf Kutsch

**Affiliations:** ^1^ Department of Microbiology, University of Alabama at Birmingham, Birmingham, Alabama, USA; ^2^ Department of Medicine, University of Alabama at Birmingham, Birmingham, Alabama, USA; ^3^ Department of Medical Microbiology and Infection Control, VU University Medical Center, Amsterdam, Netherlands

**Keywords:** HIV-1, PPM1A, macrophages, Mycobacterium tuberculosis, persistent infection, Immunology and Microbiology Section, Immune response, Immunity

## Abstract

Co-infection with HIV-1 and *Mycobacterium tuberculosis* (*Mtb*) is a major public health issue. While some research has described how each pathogen accelerates the course of infection of the other pathogen by compromising the immune system, very little is known about the molecular biology of HIV-1/*Mtb* co-infection at the host cell level. This is somewhat surprising, as both pathogens are known to replicate and persist in macrophages. We here identify Protein Phosphatase, Mg^2+^/Mn^2+^-dependent 1A (PPM1A) as a molecular link between *Mtb* infection and increased HIV-1 susceptibility of macrophages. We demonstrate that both *Mtb* and HIV-1 infection induce the expression of PPM1A in primary human monocyte/macrophages and THP-1 cells. Genetic manipulation studies revealed that increased PPMA1 expression rendered THP-1 cells highly susceptible to HIV-1 infection, while depletion of PPM1A rendered them relatively resistant to HIV-1 infection. At the same time, increased PPM1A expression abrogated the ability of THP-1 cells to respond to relevant bacterial stimuli with a proper cytokine/chemokine secretion response, blocked their chemotactic response and impaired their ability to phagocytose bacteria. These data suggest that PPM1A, which had previously been shown to play a role in the antiviral response to Herpes Simplex virus infection, also governs the antibacterial response of macrophages to bacteria, or at least to *Mtb* infection. PPM1A thus seems to play a central role in the innate immune response of macrophages, implying that host directed therapies targeting PPM1A could be highly beneficial, in particular for HIV/*Mtb* co-infected patients.

## INTRODUCTION

As gatekeepers of the innate immunity, macrophages contribute significantly to Human Immunodeficiency Virus-1 (HIV-1) pathogenesis throughout the course of viral infection. The crucial role of macrophages during HIV-1 infection has been conclusively demonstrated [1, 2]. For SIV infection of macaques, macrophages are sufficient to drive high level, systemic infection even in the complete absence of CD4^+^ T cells [3]. Further, it is becoming increasingly clear that cells of the monocyte/macrophage lineage contribute to the viral reservoir that persists despite antiretroviral treatment, as persistently HIV-1 infected macrophages migrate into the brain [4], gut [5], lymphoid tissues [6], or even adipose tissue [7]. Nevertheless, compared to our vast understanding of HIV-1 pathogenesis in T cells, relatively little is known about HIV-1 infection in macrophages. This is particularly true in the setting of HIV-1 and *Mycobacterium tuberculosis* (*Mtb*) co-infection.

HIV-1 and *Mtb* co-infections have emerged as a global health threat as the morbidity and mortality associated with HIV-1/*Mtb* co-infections is greatly exacerbated compared to infections with each individual pathogen alone [8]. As HIV-1 also targets macrophages, the principal host cell for *Mtb*, it directly interferes with the ability of these innate immune cells to control *Mtb* infection. HIV-1 infection of macrophages affects proper cytokine production in response to *Mtb* infection [9, 10], and prevents phagosome acidification, which is essential to kill intracellular *Mtb* [11, 12].

While most studies have looked at how HIV-1 infection impacts tuberculosis (TB) pathogenesis, fewer studies have investigated how *Mtb* infection impacts HIV-1 pathogenesis. These studies mostly focus on the detrimental effect of *Mtb* infection on the HIV-1-specific immune response [13-15]. HIV-1 replication was shown to be increased at sites of *Mtb* infection in the lung [16], in acutely *Mtb*-infected macrophages [17, 18], and in alveolar macrophages from HIV-1 patients infected with *Mtb ex vivo* [19]. It has been shown that *Mtb* can promote HIV-1 infection by increasing the expression of CXCR4 and CCR5, the two HIV-1 co-receptors [20] and increase the susceptibility of CD4^+^ T cells to HIV-1 infection through a TLR2-mediated pathway [21]. It has also been reported that increased TNF-α production following *Mtb* infection can activate HIV-1 replication in macrophages [20, 22]. Others suggested a decrease in viral replication as a consequence of *Mtb* co-infection [23]. Much of this research is descriptive in nature and very little is known about the molecular biology at the host cell interface of these two pathogens during co-infection of macrophages [24-26]. A more detailed understanding of the biomolecular changes in *Mtb*-infected macrophages during co-infection with HIV-1 would possibly allow for the identification of novel treatment strategies.

Here, we demonstrate how *Mtb* infection boosted the expression of Protein Phosphatase, Mg^2+^/Mn^2+^ Dependent 1A (PPM1A) in macrophages, a phenotype that undermined the intrinsic antiviral cellular response to promote HIV-1 infection. A role for PPM1A in the anti-HIV-1 response has not been previously demonstrated in macrophages, but is consistent with a report of its role in antiviral signaling during Herpes Simplex Virus (HSV) infections [27]. We further show that HIV-1 infection of macrophages directly up-regulated PPM1A expression, suggesting that virus-mediated PPM1A up-regulation would be a previously undescribed viral escape mechanism. Lastly, we demonstrate that PPM1A not only controls the antiviral response, but also controls the antibacterial response of macrophages against *Mtb* infection. Our results introduce PPM1A as a protein that is central to the general innate immune response of macrophages. Specifically in the context of HIV-1/*Mtb* co-infection, our results suggest that infection by either pathogen will enforce phenotypic biomolecular changes that render macrophages into highly vulnerable targets for HIV-1 or *Mtb* infection, a process that is linked at the molecular level by the pathogen-induced up-regulation of PPM1A expression.

## RESULTS

### A model of persistent *Mtb* infection in THP-1 monocytes/macrophages

To increase our knowledge on the molecular biology of HIV-1/*Mtb* co-infection at the macrophage host cell level, we would need an experimental model that (i) supports infection with either pathogen, (ii) produces sufficient and defined cell material and (iii) must be amenable to genetic manipulations. *In vivo*, monocytes/macrophages aggregate around the site of *Mtb* infection in order to eliminate or contain the pathogens, a process that finally produces highly complex granuloma structures that involve many different host cell types. Interestingly, in HIV-1/*Mtb* co-infected patients, this process seems impaired [28] and at the same time, HIV-1 infection was shown to be increased at sites of *Mtb* infection in the lung [16]. Standard experimental protocols that use differentiated macrophages do not reproduce the formation of *Mtb*/macrophage aggregates, however, it is these aggregate structures or granulomas that have been reported to host persistent *Mtb* infection and that serve as the interface for *Mtb* and HIV-1 co-infection [28-30].

**Figure 1:**
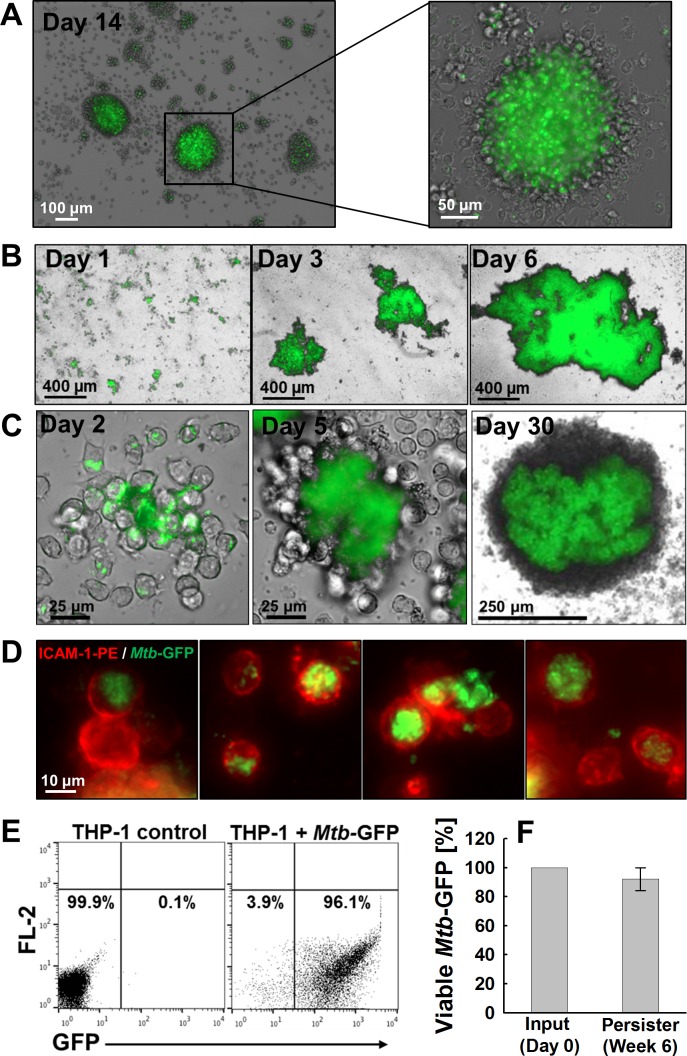
Persistent *M. tuberculosis* **infection in different monocyte/macrophage models.** Infection of PBMC derived primary human monocytes with *Mtb*-GFP at an MOI of **A.** 1 or **B.** 10 induced *Mtb*/macrophage aggregate formation as shown by representative merged bright field and GFP channel images. **C.** Dynamics of aggregate formation by THP-1 monocytes infected with *Mtb*-GFP were imaged and representative bright field and GFP merged images are shown on day 2, day 5, and day 30 post infection. **D.** Establishment of long-term persistently *Mtb*-infected THP-1 macrophages are shown as representative fluorescence images of single cell macrophages persistently infected with variable *Mtb* burden (green) stained with α-ICAM-1-PE antibody (red) at 6 weeks post infection. **E.** Single cell macrophages in (D) were analyzed by flow cytometry to measure the frequency of persistently *Mtb*-infected cells using GFP as a surrogate marker for *Mtb*-infected macrophages. **F.** Intracellular survival of *Mtb* extracted from persistently infected macrophages at 6 weeks post infection were enumerated by CFU plating and normalized to initial input as 100%.

We thus used recent work that demonstrated the formation of granuloma structures in peripheral blood mononuclear cells (PBMC) infected with *Mtb* to guide our experiments [31, 32]. We reasoned that direct *Mtb* infection of primary monocytes with a high MOI would increase the likelihood of *Mtb*/macrophage aggregate formation. Infections were performed with an auxotrophic, *gfp*-expressing *Mtb* strain (*Mtb* mc^2^6206; H37Rv derivative, Δ*panCD*, Δ*leuCD*) chosen to allow experiments under BSL2 conditions [33]. *Mtb*-infection induced monocyte differentiation indeed resulted in the formation of extensive *Mtb*/macrophage aggregate structures that reached sizes of up to 2 mm (Figure 1A, 1B). Over an 8 days observation period, key cytokines expressed by macrophages during *Mtb* infection such as IL-1α/β, TNF-α, IL-6 and IL-10 showed dynamic expression profiles consistent with previously published literature (Supplementary Figure 1A-1E) [34, 35]. *Mtb* extracted from the formed *Mtb*/macrophage aggregates on day 8 post infection was found to be viable (Supplementary Figure 1F), which is consistent with the finding that *Mtb* can persist in *Mtb*/aggregates or granuloma structures [36]. However, to study how persistent *Mtb* infection would alter macrophage signaling pathways using a systems biology approach such as kinome array analysis, we would need a highly pure population of *Mtb*-infected macrophages, free of uninfected bystander cells. This was not enabled by this model, as the formed *Mtb*/macrophage aggregates were extremely stable and did not allow for the extraction of single, viable macrophages. Thus, we next transferred this high MOI infection approach to a suitable monocyte/macrophage cell line.

Macrophage infection models for *Mtb* studies have extensively utilized THP-1 cells [37-39], which have also been widely used in HIV-1 research [40-42], and proven highly useful in the discovery of macrophage restriction factors to HIV-1 [43, 44]. *Mtb* infection-mediated differentiation of THP-1 monocytes induced the formation of *Mtb*/macrophage aggregate structures (Figure 1C) in which the activation state of the macrophages was apparent by strongly increased levels of ICAM-1 expression (Supplementary Figure 2A, 2B). ICAM-1 is an adhesion molecule known to be up-regulated in mature or activated monocytes/macrophages [45, 46] and has been shown to play an essential role in *in vivo* granuloma formation [47, 48]. Under optimal aggregate-forming conditions, THP-1 cells initially completely phagocytosed the bacteria (Supplementary Figure 2C, 2D), and then formed high density cell aggregates containing a core of dead cell debris and heavily infected macrophages that increased in size over time (Figure 1C). Transition of the long-term infection culture through the aggregation phase again recapitulated many reported features of *Mtb* infection, such as the production of several key cytokines (TNF-α, IL-1α/β, IL-10; Supplementary Figure 3A-3D) and chemokines (IL-8, MIP-1α/β, and MCP-1; Supplementary Figure 3E-3H) involved in host cell responses to *Mtb* infection. Importantly and different from the primary monocyte system, after 4 - 6 weeks, large amounts of single, persistently *Mtb*-infected THP-1 cells could be retrieved from the culture (Figure 1D, 1E). Flow cytometric analysis demonstrated that 6 weeks post infection, these persistently *Mtb*-infected THP-1 cells comprised more than 90% of the entire THP-1 population (Figure 1E). Despite being intracellularly contained, *Mtb* extracted from these persistently infected THP-1 cells was found viable, demonstrating that this *Mtb*/macrophage aggregate infection model would reproduce reports that *Mtb* can viably persist in macrophages (Figure 1E, 1F) [49, 50].

### Persistently *Mtb*-infected macrophages exhibit a massively altered kinome signature

To probe for changes in the cell signaling network that *Mtb* enforces in the host macrophage to establish persistent infection, we employed kinome array analysis, a systems biology approach used to simultaneously profile the expression levels of hundreds of protein kinases, phosphatases and other regulatory proteins within a defined cell population. The utilized Kinexus antibody-based kinome arrays provide information on changes in the expression or phosphorylation state of 309 protein kinases, 38 protein phosphatases, 37 stress response proteins, 24 transcription factors, and 109 proteins involved in other signaling pathways.

**Figure 2:**
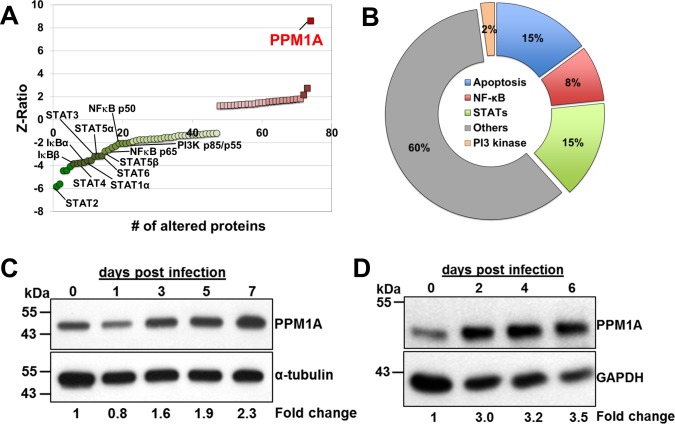
PPM1A expression is up-regulated in macrophages during *M. tuberculosis* **infection.** Cell lysates of persistently *Mtb*-infected THP-1 macrophages (Figure 1D, 1E) and the corresponding non-infected controls were used for kinome analysis using Kinexus antibody arrays. **A.** The 74 differentially expressed signaling proteins were plotted ranked according to their Z-ratio, which indicates statistically significant up- (red) or down-regulation effects (green). The most prominent signal was that of Protein Phosphatase, Mg^2+^/Mn^2+^-dependent 1A (PPM1A) expression (Z-ratio = 8.6). **B.** Pie-chart showing the relative numbers of identified altered signaling proteins and their association with different functional categories or pathways. **C.** PPM1A protein expression levels from lysates of *Mtb*-infected THP-1 cells or **D.** primary human monocytes at various days post *Mtb*-infection were analyzed by Western blotting. Densitometry analysis was performed by ImageJ to quantify PPM1A band intensities as normalized to α-tubulin or GAPDH, and fold changes relative to day 0 post infection are reported.

Antibody arrays revealed a total of 74 changes detected at the protein expression level or the phosphorylation state in the lysates of THP-1 cells persistently infected with *Mtb* when compared to non-infected THP-1 cells (Supplementary Table 1). 56 spots indicated differences in protein levels and 18 in phosphorylation states (Supplementary Table 1). Among these, 37 kinases, 17 proteins involved in transcription regulation, 3 phosphatases, and 23 proteins involved in other signaling pathways were found altered (Figure 2A and Supplementary Table 1). Interestingly, of the 74 spots, 47 (64%) indicated a reduction in protein levels or phosphorylation state of the respective signaling protein. Moreover, 22 of the 47 signaling proteins showed strong down-regulation of expression (Z-ratio < −2), while only 3 proteins were strongly up-regulated (Z-ratio of > 2) (Figure 2A). Of the 47 signaling proteins with reduced expression/activity, 7 were associated with the STAT pathway, 7 with the apoptosis pathway, 4 with the NF-κB pathway, and 1 with the PI3K pathway, confirming that the model faithfully reproduces findings previously published by others (Figure 2B) [51-55].

### PPM1A levels are elevated in *M. tuberculosis* infected human monocyte/macrophages

The most interesting individual signal on the kinome array representing a possible link to HIV-1/*Mtb* co-infection was the up-regulation of Protein Phosphatase, Mg^2+^/Mn^2+^-dependent 1A (PPM1A) expression (Z-ratio = 8.6) (Figure 2A and Supplementary Table 1). PPM1A has not been reported to play a role in bacterial infections, but through yeast two hybrid experiments, PPM1A was previously identified as an interaction partner for Stimulator of Interferon Genes (STING) and linked to the cellular innate antiviral response against HSV infection by antagonizing the type I interferon response [27]. Seeking to identify biomolecular processes that would link *Mtb* and HIV-1 infection at the interface of their common host cell, we sought to further investigate a possible role for PPM1A expression in the context of *Mtb* and HIV-1 co-infection.

Like with most systems biology methods, kinome array analysis is best utilized as a hypothesis-generating tool, and results obtained by this method require further experimental confirmation. We thus first confirmed PPM1A protein regulation in monocytes/macrophages infected by *Mtb*. For this purpose, we infected THP-1 cells and primary monocytes with *Mtb* under the same experimental conditions as described above, and quantified the expression of PPM1A over time by Western blot analysis. In these experiments, PPM1A protein levels in *Mtb*-infected THP-1 cells and in primary monocyte derived macrophages were indeed found up-regulated in a kinetic manner (Figure 2C, 2D).

### PPM1A expression levels determine macrophage susceptibility to HIV-1 infection

**Figure 3:**
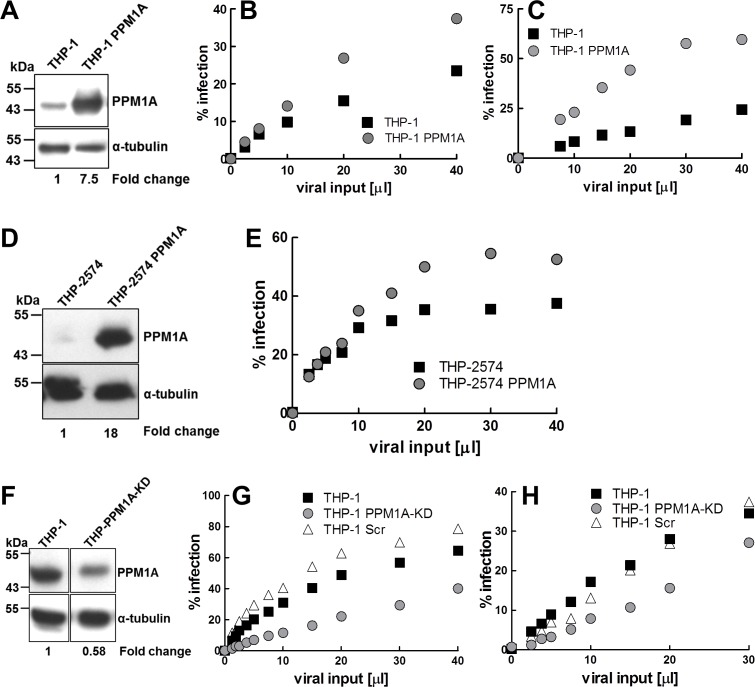
PPM1A expression levels regulate macrophage susceptibility to HIV-1 infection **A.** Lysates from THP-1 and THP-PPM1A cells were analyzed by Western blotting to quantify PPM1A expression. **B.** THP-1 or THP-PPM1A cells were infected with a VSV-G-pseudotyped HIV-GFP reporter vector using increasing amounts of viral input material adjusted to obtain maximum infection levels in THP-1 cells of ∼20%. 2 days post infection, the percentage of infected cells was determined by flow cytometry, as indicated by GFP^+^ events. **C.** THP-1 or THP-PPM1A cells were infected with HIV-1 WEAU using increasing amounts of viral input material adjusted to obtain maximum infection levels in THP-1 cells of ∼20%, as to allow to measure a possible increase in infection in THP-PPM1A cells. 2 days post infection, cells were intracellularly stained for HIV-1 p24-Gag protein expression, and the percentage of infected cells was determined by flow cytometry. **D.** Western blot analysis of PPM1A expression in THP2574 or THP2574-PPM1A reporter cells. **E.** THP2574 or THP2574-PPM1A reporter cells were infected with HIV-1 WEAU using increasing amounts of viral input. 3 days post infection, the amount of infected cells were determined by flow cytometry as indicated by GFP^+^ events. **F.** CRISPR-Cas9 genome editing mediated knockdown of PPM1A in a heterogeneous population of THP-1 cells (THP-PPM1A-KD) was analyzed by Western blotting. In the THP-PPM1A-KD population, a ∼50% reduction in PPM1A levels was achieved as quantified by densitometry. THP-1, THP-1 scrambled sgRNA control (THP-1 Scr), or THP-PPM1A-KD cells were infected with **G.** HIV-GFP or **H**. HIV-1 WEAU using increasing amounts of viral input. 2 days post infection, cells were analyzed by flow cytometry to determine the percentage of actively infected cells as indicated by GFP^+^ cells for HIV-GFP vector transductions or the percentage of p24-Gag expressing cells for HIV-1 WEAU infections, respectively.

Given the reported link between PPM1A levels and the antiviral response in HSV infection [27], we next tested whether PPM1A up-regulation in monocytes/macrophages, as induced by *Mtb* infection, would actually affect HIV-1 infection. For this purpose, we generated a PPM1A overexpressing THP-1 cell population (THP-PPM1A). The achieved PPM1A overexpression levels (Figure 3A) were comparable to the PPM1A levels that were induced during *Mtb* infection (Figure 2D). Importantly, PPM1A overexpression had no effect on general cell viability and proliferation (Supplementary Figure 4A), or on CD4, CXCR4 or CCR5 expression, the receptor/co-receptors for HIV-1 (Supplementary Figure 4B).

If PPM1A would act to disable the cellular innate antiviral response in a manner similar to its role in HSV infection, we would expect to see a post-entry effect on HIV-1 infection [27]. We thus initially used a VSV-G pseudotyped HIV-GFP vector [56] to bypass HIV-1 specific entry mechanisms and to directly assess effects of PPM1A expression on post-entry macrophage susceptibility. For this specific purpose, HIV-GFP reporter vectors are a reasonable choice since there is no reported evidence that these vectors would differ from relevant subtype B clinical isolates at the level of LTR function or other post entry functionalities. Consistent with a potential role of PPM1A in the control of cellular antiviral response, infection with HIV-GFP vectors, over a wide range of infection levels, resulted in two-fold higher levels of active infection events in THP-PPM1A cells than in THP-1 control cells (Figure 3B).

Titration of HIV-1 WEAU, a clinical HIV-1 isolate derived from a patient during acute infection [57], on either THP-1 cells or THP-PPM1A cells confirmed that PPM1A overexpression rendered macrophages more susceptible to HIV-1 infection. Over a range of viral input levels, PPM1A overexpression tripled the achievable infection levels (Figure 3C). In these initial experiments, the percentage of HIV-1 infected cells was determined by intracellular antibody staining for HIV-1 Gag p24 followed by flow cytometric analysis. However, macrophages have been reported to also phagocytose viral particles or proteins, which could potentially confound these results, in the event that parts of the detected p24 expression could arise from particle uptake rather than from infection mediated *de novo* production of HIV-1. To exclude this possibility, we generated a THP-based GFP reporter cell population that would allow us to directly measure active HIV-1 infection with clinical isolates using LTR-driven GFP expression as a surrogate marker. GFP expression in these reporter cells could not be produced by viral particle uptake, but only by active infection events that would produce HIV-1 Tat to trans-activate the LTR-GFP reporter (see Material and Methods). The obtained THP2574 reporter cell population was then retrovirally transduced to overexpress PPM1A (Figure 3D). HIV-1 WEAU was then titrated on THP2574 and THP2574-PPM1A cell populations, and infection levels were determined as %GFP-positive cells using flow cytometric analysis (Figure 3E). The results confirmed our initial findings in THP-1 cells and clearly demonstrated that PPM1A expression rendered macrophages more susceptible to HIV-1 infection.

If up-regulation of PPM1A could undermine the antiviral defense against HIV-1 infection, we reasoned that depletion of PPM1A would result in an opposing effect and would reduce macrophage susceptibility to HIV-1 infection. Accordingly, knockdown of PPM1A expression allowed the cellular innate antiviral response of macrophages to better control HIV-1 infection. Within a population of THP-1 cells, PPM1A-specific CRISPR/Cas9-mediated gene knockout would result in a ∼50% knockdown at the protein level as determined by Western blot (THP-PPM1A-KD; Figure 3F). A control cell population was generated using a CRISPR/Cas9 vector containing scrambled sgRNA (THP-Scr). Again, to limit the study on post-entry events, we infected THP-1, THP-Scr and THP-1 PPM1A-KD cells with the HIV-GFP reporter vector to assess the level of achievable active HIV-1 infection. Consistent with the data that PPM1A overexpression would increase susceptibility to HIV-1, PPM1A knockdown decreased susceptibility to HIV-GFP (Figure 3G). In extension, achievable HIV-1 WEAU infection levels of THP-PPM1A-KD cells were found reduced when compared to infection of naïve THP-1 cells (Figure 3H), a result consistent with the idea that PPM1A would control the innate antiviral response of macrophages.

### PPM1A expression levels have no effect on Tat-dependent or independent HIV-1 LTR activity

To exclude the possibility that PPM1A would control macrophage susceptibility to HIV-1 infection by affecting HIV-1 LTR promoter activity, we assessed the effect of PPM1A on Tat-dependent or independent HIV-1 LTR activity. To determine whether baseline LTR activity or responsiveness in the absence of Tat would be altered in the presence of high PPM1A protein levels, we stimulated THP2574 and THP2574-PPM1A reporter cells with the PKC/NF-κB activating phorbol ester PMA and measured Tat-independent LTR-induction by using GFP expression as a surrogate marker. No relevant difference in induced GFP expression levels as a function of PMA concentration could be detected (Supplementary Figure 5A), suggesting that PPM1A would not affect baseline or NF-κB activation induced HIV-1 LTR activity. To test whether PPM1A would affect Tat-induced LTR-activity, we retrovirally transduced THP2574 and THP2574-PPM1A cells with a MSCV-Tat expression vector. Again, as shown in Supplementary Figure 5B, no relevant difference in the level of Tat-induced, LTR-driven GFP expression was observed, suggesting that PPM1A acted by interference with the innate antiviral response of macrophages [27], and not by interfering with LTR-driven HIV-1 expression.

### HIV-1 infection up-regulates PPM1A expression in macrophages

**Figure 4:**
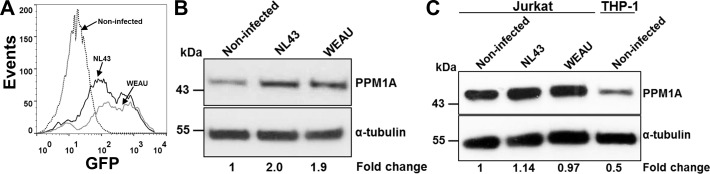
HIV-1 infection directly induces PPM1A expression in macrophages **A.** Infection levels of HIV-1 NL43 and HIV-1 WEAU infected THP2574 reporter cells used for Western blot in (B) as assessed by flow cytometric analysis using GFP expression as a surrogate marker of active HIV-1 infection. **B.** Western blot analysis of PPM1A levels in THP2574 cells following infection with HIV-1 NL43 or HIV-1 WEAU for 24 h. **C.** Western blot analysis of PPM1A levels in Jurkat T-cells following infection with HIV-1 NL43 or HIV-1 WEAU for 24 h in comparison to baseline PPM1A expression in the monocytic THP-1 cells. Densitometry analysis in (B) and (C) was performed by ImageJ to quantify PPM1A band intensities as normalized to α-tubulin, and fold changes are expressed relative to the non-infected cells.

PPM1A was reported to be recruited to the STING complex in response to HSV-1 infection [27], a mechanism that would allow HSV-1 to escape the antiviral response mediated through the production of type I interferons. However, a possible regulation of PPM1A expression by viral infection was not examined in this study. We thus next investigated whether HIV-1 infection would directly alter PPM1A expression in macrophages. This would establish the idea that HIV-1 (or for that matter other viruses) could have evolved a viral escape mechanism that would inactivate the innate antiviral response in macrophages by infection-induced up-regulation of PPM1A. For this purpose, we infected THP2574 cells with either HIV-1 NL43 or HIV-1 WEAU. In either case, correlating with an infection rate of 56% or 70% for HIV-1 NL43 or WEAU, respectively (Figure 4A), a ∼2-fold increase in PPM1A protein levels was observed 24 h post infection (Figure 4B). As our HIV-1 infection data in PPM1A overexpressing THP-1 cells suggest that PPM1A up-regulation is likely to interfere with viral silencing mechanisms, HIV-1 may have indeed evolved to undermine a cellular innate antiviral mechanism in an attempt to counteract viral silencing. It is also important to note that PPM1A must play a completely different role in HIV-1 infection of macrophages and T cells. PPM1A had been previously investigated for a potential role in HIV-1 infection of T cells. Different from monocyte/macrophages, T cells express high levels of PPM1A, which is not regulated upon T cell activation [58]. We confirm this finding and demonstrate that PPM1A levels in T cells are also not regulated by HIV-1 infection (Figure 4C). Knockdown of PPM1A in T cells was reported to result in a decrease in HIV-1 expression [58], a finding that was attributed to the interaction of PPM1A with the pTEFb complex and subsequent effects on RNAP II processivity, which clearly was not the case for macrophages, where PPM1A expression was positively correlated with HIV-1 infection.

### PPM1A up-regulation impairs the antibacterial response of macrophages

Given the observed PPM1A up-regulation following HIV-1 or *Mtb* infection, we investigated whether PPM1A expression would not only affect the antiviral response, but also affect the antibacterial response.

The kinome data depicted in Figure 2A and 2B (and Supplementary Table 1), showing down-regulation of several STAT family members, PI3 kinase, and proteins involved in the NFκB signaling pathways, strongly suggest that the functionality of persistently *Mtb*-infected macrophages would be severely impaired. A protein-protein interaction network (PIN) based on altered signaling proteins as seed nodes provides a graphical description of this point (Supplementary Figure 6). This PIN would predict massive impairments to the ability of macrophages to respond to relevant external stimulation.

Cellular parameters important for general macrophage functionality appeared phenotypically identical in PPM1A overexpressing cells as we observed no differences relative to the parent strain in terms of viability, proliferation (Supplementary Figure 4A), and cell surface expression of key molecules such as CD4, CD14, CD16, ICAM-1, HLA-DR, or TLR5 at baseline and following IFN-γ or LPS-stimulation (Supplementary Figure 7).

**Figure 5:**
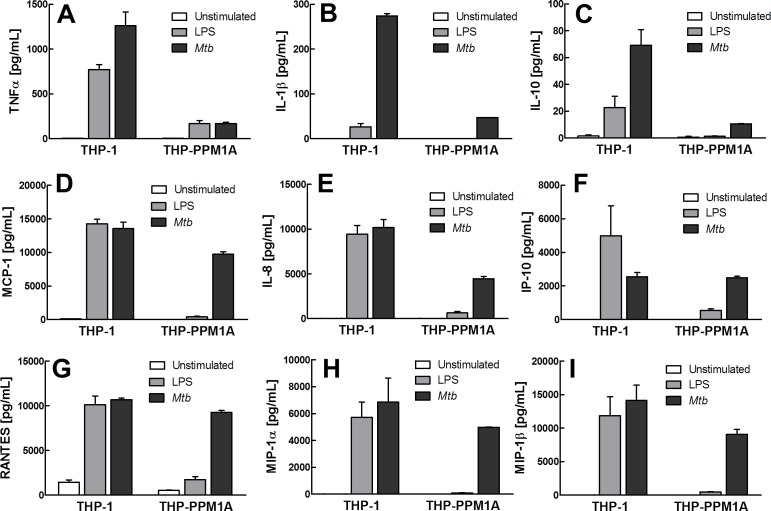
PPM1A negatively regulates monocyte response to bacterial stimuli **A.**-**I.** Culture supernatant from unstimulated, LPS-stimulated (100 ng/mL) or *Mtb*-exposed (MOI 50) THP-1 or THP-PPM1A cells were collected at 24 h and analyzed by Milliplex assays to quantify levels of (A) TNFα, (B) IL-1β, (C) IL-10, (D) MCP-1, (E) IL-8, (F) IP-10, (G) RANTES, (H) MIP-1α, and (I) MIP-1β. Results are expressed as the mean ± standard deviation of three independent experiments.

**Figure 6:**
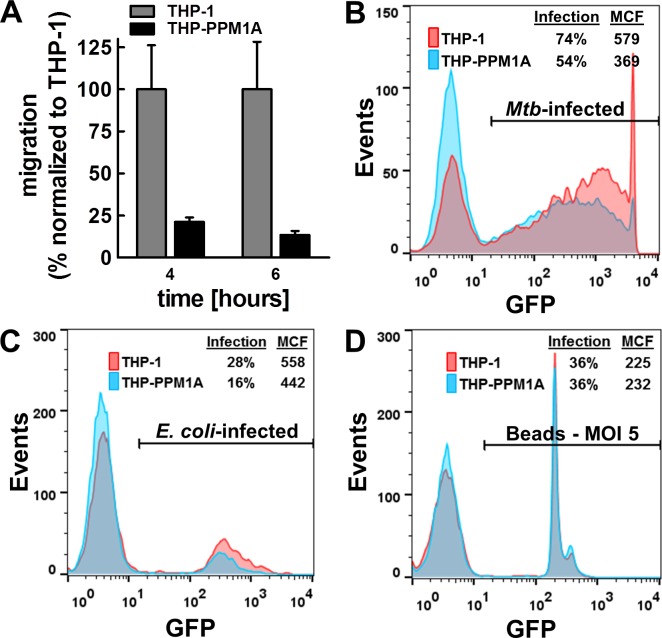
Elevated PPM1A levels impair macrophage functions **A.** The number of migrated cells (THP-1 or THP-PPM1A) through a 5 μm transwell membrane towards a mixture of chemokines (supernatant harvested from *Mtb*-infected THP-1 cells; Supplementary Figure 3) was determined by counting the number of cells in the lower chamber by flow cytometry. Results are normalized to THP-1 cell migration events as 100% and expressed as the mean ± standard deviation of three independent experiments. **B.** THP-1 or THP-PPM1A cells exposed to *Mtb*-GFP (MOI 50) for 24 h were analyzed by flow cytometry to quantify the level of phagocytosis using GFP as a marker for internalized *Mtb*. **C.** THP-1 or THP-PPM1A cells were exposed to *gfp*-expressing *E. coli* (MOI 10) or **D.** 3 μm rainbow polystyrene beads (MOI 5) for 1 h to allow for phagocytosis. The GFP signal indicates cells that have taken up *E. coli* or beads

However, consistent with the kinome profile, cytokine and chemokine production following stimulation by LPS or exposure to *Mtb* was massively impaired in THP-PPM1A cells (Figure 5A-5I). Previously reported key cytokine and chemokine responses to *Mtb* infection, such as production of the inflammatory cytokines TNF-α [59], IL-1β [60, 61], the anti-inflammatory cytokine IL-10, or the chemokine IL-8 [62], were diminished in THP-PPM1A cells (Figure 5). Other chemokines that were not induced by LPS, but could be induced by *Mtb* were MCP-1, IP-10, RANTES, MIP-1α and MIP-1β (Figure 5). PPM1A overexpression not only affected the expression of chemokines, but also affected the ability of monocyte/macrophages to migrate in response to chemokines. While THP-1 cells would efficiently migrate from the apical insert into the basolateral well of a transwell setup, where a chemokine signal was presented in the form of supernatants harvested from *Mtb*-infected THP-1 monocytes (for chemokine composition see Figure 5 and Supplementary Figure 3), THP-PPM1A cell migration was significantly impaired as indicated by ∼75% less migration relative to THP-1 cells that migrated towards the chemotactic signal (Figure 6A).

Lastly, we tested the ability of monocyte/macrophages to properly phagocytose foreign particles as a function of PPM1A expression. We demonstrated that THP-PPM1A cells exhibited reduced uptake capacity for bacteria (Figure 6B, 6C). *Mtb* uptake by THP-PPM1A cells was reduced by ∼30% when compared to THP-1 cells (Figure 6B), while uptake of *E. coli* was ∼40% less efficient in THP-PPM1A cells (Figure 6C). Interestingly, uptake of 3 μm polystyrene beads was unaffected by PPM1A expression levels (Figure 6D), suggesting that the phagocytosis defect may be driven by specific receptor functionality. Similar results were obtained in PMA differentiated THP-1 cells (data not shown) suggesting that the enhanced phagocytic capability of differentiated THP-1 cells [63] could not restore PPM1A mediated impairment of phagocytosis. Collectively, these data suggest a link between PPM1A and essential signaling pathways that govern the antibacterial response of macrophages.

## DISCUSSION

Despite clear evidence on the important role of monocytes and macrophages during HIV-1 infection [2, 6], HIV-1 pathogenesis research is mostly focused on T cells. This is even true in the setting of HIV-1/*Mtb* co-infection, where macrophages play an important role in controlling *Mtb* infection [64], but also serve as the common host cell to both pathogens. In this study, we describe that infection with either pathogen up-regulated the expression of Protein Phosphatase, Mg^2+^/Mn^2+^ Dependent 1A (PPM1A), which we identify as a molecule that directly links the antiviral and the antibacterial response of macrophages. PPM1A up-regulation rendered macrophages highly susceptible to HIV-1 infection, and inert to bacterial stimuli.

In a recent report by Li *et al*. [27], PPM1A was shown to negatively regulate antiviral signaling by dephosphorylating, and thereby inactivating STING and TANK-binding kinase 1 (TBK1), which are both necessary to mount an antiviral response through production of type I interferons. In this study, overexpression of PPM1A inhibited STING mediated antiviral signaling, and an shRNA mediated knockdown of PPM1A in THP-1 cells resulted in an increased antiviral response to HSV-1 as indicated by increased interferon-β production [27]. In extension of the work of Li *et al*. [27], we show that overexpression of PPM1A in THP-1 cells increased susceptibility to HIV-1 infection (Figure 3A-3E). Conversely, knockdown of PPM1A expression reduced the susceptibility of macrophages to HIV-1 infection (Figure 3F-3G). A particularly interesting aspect of the presented data is that HIV-1 infection by itself increased PPM1A expression levels (Figure 4). Given the finding that regulation of PPM1A expression plays a critical role in the host response to HIV-1 infections, the documented ability of HIV-1 to up-regulate PPM1A expression is the first report that viral infections can affect PPM1A expression levels, and also suggests that viruses, and certainly HIV-1, may have developed the ability to escape the cellular innate antiviral response in macrophages by up-regulating PPM1A.

The role of PPM1A in the context of HIV-1 infection had previously been studied in T cells, where PPM1A expression was found to inhibit HIV-1 expression [58]. In this report, Budhiraja *et al*. demonstrated high levels of PPM1A expression in resting CD4^+^ T cells, that were not further up-regulated upon T cell activation. While we confirm that T cells express high baseline levels of PPM1A and demonstrate that HIV-1 infection does not affect PPM1A expression levels in T cells (Figure 4C), we find relatively low basal levels of PPM1A in monocytes, which are then up-regulated upon infection by *Mtb* or HIV-1 (Figures 2C, 2D and 4B, 4C). Also, PPM1A expression in T cells obviously was important for RNAP II processivity [58], very different from its role in the antiviral response of monocyte/macrophages suggesting that PPM1A signaling and/or regulation differs in monocytes/macrophages and T cells.

*Mtb*-infection induced regulation of PPM1A expression should be of general interest to research on bacterial infections, as we here demonstrate that PPM1A up-regulation not only subverts the cellular innate antiviral response of macrophages, but also drives macrophages into an immune paralyzed state (Figure 2A, 2B; Supplementary Figure 6). Infection induced PPM1A up-regulation abrogated the ability of macrophages to respond to bacterial stimuli such as LPS with efficient cytokine or chemokine production (Figure 5), abrogated their ability to migrate in response to a chemotactic stimulus (Figure 6A), and impaired the ability of macrophages to efficiently phagocytose bacteria (Figure 6B-6D). The implications of these findings likely extend beyond the immediate scope of *Mtb* research and apply to other intracellular bacterial pathogens where the host macrophage would play an integral role in control of pathogenesis.

In the setting of HIV-1/*Mtb* co-infection, *Mtb* infection-mediated up-regulation of PPM1A followed by increased HIV-1 susceptibility results in a scenario that could provide the underlying molecular mechanism to explain previous reports that have shown increased HIV-1 replication at sites of *Mtb* infection in the lung [16], and in acutely *Mtb*-infected macrophages [17, 18].

Collectively, our findings establish PPM1A as a central component of both, the antiviral and antibacterial response of macrophages, at least for HIV-1 and *Mtb* infection. Specifically in the context of HIV-1/*Mtb* co-infection, our data show that PPM1A plays a critical role at the interface of HIV-1/*Mtb* co-infection. *Mtb* infection-induced PPM1A up-regulation in macrophages would act to undermine a cellular antiviral defense mechanism to boost HIV-1 infection. Conversely, up-regulation of PPM1A expression in response to HIV-1 infection would be detrimental for the antibacterial response to *Mtb* infection. The significance of our findings rests with the possibility that controlling the regulatory function of PPM1A by drug intervention has the potential to interrupt the notorious amplification cycle of HIV-1/*Mtb* co-infection. Our data suggest that inhibition of PPM1A could prevent or reverse the establishment of persistent *Mtb* infection and simultaneously inhibit HIV-1 infection of macrophages. Unfortunately, no pharmaceutical PPM1A inhibitors are available at the time. Lastly, our data also add to the evolving concept that exposure to infectious conditions trigger permanent phenotypic changes in host cells that can have far reaching consequences for the course of pathogenesis, likely not limited to HIV-1/*Mtb* co-infection [65].

## MATERIALS AND METHODS

### Cell culture, reagents, and antibodies

THP-1 monocytes (ATCC TIB-202), Jurkat T cells (ATCC TIB-152) and primary monocytes were maintained in RPMI 1640 medium supplemented with 2 mM L-glutamine and 10% heat-inactivated fetal bovine serum (FBS) at 37°C in a humidified atmosphere of 5% CO_2_. Human PBMCs were isolated from buffy coats by the Ficoll-Paque density centrifugation method. Monocytes were enriched by positive selection using anti-CD14 mAb-coated microbeads from Miltenyi Biotec (San Diego, CA) according to manufacturer's protocol. Fetal bovine serum was obtained from Life Technologies (Grand Island, NY). The phorbol ester 13-phorbol-12-myristate acetate (PMA), puromycin and LPS were purchased from Sigma (St. Louis, MO). Recombinant human Interferon-gamma was purchased from Genscript (Piscataway, NJ). PE-conjugated antibodies to human CD4, CD14, HLA-DR, and ICAM-1 were purchased from BD Biosciences (San Diego, CA). Anti-CD16-PE and anti-PPM1A antibodies were purchased from Thermo Scientific (Rockford, IL). Anti-TLR5-PE antibody was purchased from ABCAM (Cambridge, MA). Monoclonal mouse antibodies to GAPDH and α-tubulin were purchased from Santa Cruz (Dallas, Texas) and Cell Signaling (Danvers, MA), respectively.

### THP2574 reporter cells

THP2574 reporter cells were generated by transducing THP-1 cells with a lentiviral HIV-1 reporter construct (p2574) in which an HIV-1 subtype B LTR controls the expression of green fluorescent protein (GFP) [66]. The HIV-1 LTR and the GFP gene are separated by a 2,500-bp spacer element. Lentiviral particles were produced by transfecting 293T cells with p2574 and supplying gag-pol-rev-tat *in trans*. Vesicular stomatitis virus G (VSV-G) was used as a viral envelope protein to optimize transfection efficacy. Following lentiviral transduction of THP-1 cells, all cells that spontaneously expressed GFP were removed by cell sorting. The GFP-negative population was then activated with TNF-α to identify all cells that would harbor an inducible LTR-GFP-LTR integration event. Cells that turned GFP positive following stimulation were again selected by cell sorting. GFP expression in this population ceased after a few days, leaving a population of GFP-negative reporter cells. The amount of founder cells for this population is calculated to represent >100,000 individual integration events.

### Cell migration assay

The migration capacity of THP-1 or THP-PPM1A cells in response to chemokine exposure was assessed using 5 μm pore size PET hanging transwell inserts (Millipore) and quantified using flow cytometric analysis. Briefly, cells were suspended in the apical chamber of the transwell at 10^6^ cells/mL, and culture supernatant from THP-1 cells infected with *Mtb* for 2 days, which contain a cocktail of chemokines (see Figure 5), was added to the basolateral side of the transwell contained within a 24-well plate. Migration of cells was quantified at 4 h and 6 h by taking an aliquot of the cells that had migrated into the lower chamber and determining absolute cell counts using a Guava EasyCyte flow cytometer.

### Bacteria and plasmids

The *M. tuberculosis* H37Rv derived auxotroph strain mc^2^6206 was grown in Middlebrook 7H9 medium (Difco) supplemented with 0.2% glycerol, 0.02% Tyloxapol and 10% OADC (Remel) or on Middlebrook 7H10 plates supplemented with 0.5% glycerol and 10% OADC (Remel). Growth media of the auxotrophic *M. tuberculosis* strain was supplemented with 24 μg/ml pantothenate and 50 μg/ml L-leucine [33]. Hygromycin B was purchased from Calbiochem. The plasmid pMN437 [67] was transformed into *M. tuberculosis* mc^2^6206 to express GFP, which was used for all experiments in this study. *Escherichia coli* strain DH5α was used for cloning and infection experiments, and was routinely grown in Luria-Bertani broth at 37°C. pMSCV-Tat was constructed previously [68]. Plasmid pMSCV-PPM1A was constructed by inserting the PCR amplified *ppm1a* gene (Entrez Gene ID 5494, variant 1) from 293T-cell derived cDNA using the oligonucleotide pair 5′-gaaaCTCGAGatgggagcatttttagacaagc and 3′-tgttGTTAACttaccacatatcatctgttgatgtag, flanked by XhoI and HpaI restriction sites (capitalized), respectively, into the multiple cloning site of pMSCV-puro for retroviral expression. The resulting plasmid was sequence verified. Human PPM1A CRISPR/Cas9 knock-out plasmids were purchased from Applied Biological Materials Inc (Richmond, BC, Canada). These plasmids (pLenti-U6-sgRNA-SFFV-Cas9-2A-Puro) co-express the Cas9 protein along with the PPM1A sgRNA for lentiviral transduction. The sgRNA sequence targeting PPM1A used is 5′-GCACATACGGCTGTGAT-3′.

### Transduction

HEK 293T cells seeded at 50% confluency were transfected with retroviral or lentiviral plasmids using Fugene. Culture supernatants were harvested after 48 h and 72 h, aliquoted, and stored at −80°C. The supernatants containing retroviral particles were used to transduce THP-1 cells.

### HIV-1 strains and infection

HIV-1 infection experiments were conducted with a primary patient isolate, HIV-1 WEAU, which is primarily R5-tropic [57]. Viral stocks were prepared by transfection of the plasmids into 293T cells. Viral supernatants were then harvested 2 days post transfection, aliquoted, and stored at −80°C.

### Bacterial infection

*M. tuberculosis* mc^2^6206 or *E. coli* DH5α growing in log-phase was quantified by optical density measurement at 600 nm using the conversion of 3 × 10^8^ (*Mtb*) or 1 × 10^9^ (*E. coli*) bacteria per mL for OD 1.0. The amount of bacteria required for various MOIs were washed, resuspended in cell culture media, and added to the monocyte cultures. Monocytes and bacteria were then resuspended together to ensure even exposure of the monocytes to bacteria.

### Flow cytometry

Infection levels in cell cultures were monitored by flow cytometric (FCM) analysis of GFP expression. FCM analysis was performed on a Guava EasyCyte (Guava Technologies Inc., Billerica, MA). Data analysis was performed using Guava Express software (Guava Technologies Inc.) or FlowJo V10 software (Ashland, OR).

### Fluorescence microscopy

Imaging was performed using an Axiovert 200 microscope (Carl Zeiss) equipped with a 100x/1.4 Plan-Apochromat (Carl Zeiss). Images were recorded using an AxioCam MRc camera (Carl Zeiss) coupled to Axiovision v4.5 software (Carl Zeiss). To image *Mtb*/macrophage aggregate progression within 12-well plates, the Cytation 3 Cell Imaging Multi-Mode Reader (BioTek, Winooski, VT) fitted with 2.5x or 20x objectives and the GFP and RFP (red fluorescent protein) filter set was used.

### BioPlex analysis of cytokine and chemokine expression

Culture supernatants from LPS-stimulated or *Mtb*-infected monocytes were harvested at various time points and frozen at −80°C. Cytokine expression was determined using a MilliPlex kit for IL-1α/β, IL-8, IL-10, TNFα, MIP1α/β, IP-10, and MCP-1 (Millipore, Billerica, MA). Experiments were performed according to manufacturer's protocol and read-out was performed using a Bio-Plex 200 instrument. Data analysis was performed using Bio-Plex manager 6.0 software (Bio-Rad, Hercules, CA).

### Western blots

Cells were harvested by centrifugation, washed once with PBS, and lysed in RIPA buffer (Cell Signaling) according to the manufacturer's instructions. Protein concentration of the lysates was determined by the bicinchoninic acid (BCA) method according to the manufacturer's recommendations (Thermo Scientific). About 10 to 20 μg of protein per sample was separated on 10% Mini-Protean TGX gels (Bio-Rad) and subsequently transferred to a polyvinylidene difluoride (PVDF) membrane using an iBlot gel transfer system (Life Technologies). Western blot analysis was performed according to standard protocols. Total PPM1A, α-tubulin, or GAPDH proteins were detected with specific antibodies (see antibody section). A horseradish peroxidase-conjugated goat anti-rabbit or goat anti-mouse polyclonal antibody (Santa Cruz) was used as the secondary antibody. The blot was developed using the Western Lightning Ultra chemiluminescent substrate from Perkin Elmer, Inc., and detected in an EpiChemi3 darkroom (UVP BioImaging Systems).

### Statistical analysis

All data are expressed as the mean ± the standard deviation of at least three independent experiments. Statistical analysis was performed using the Student *t* test. Values of *p* < 0.05 were considered to be significant.

### Kinex™ antibody microarray-based analysis

To perform the Kinex™ antibody array analysis, 50 μg of lysate protein from each sample were covalently labeled with a proprietary fluorescent dye according to the manufacturer's instructions (Kinexus, Canada). After the completion of the labeling reaction, free dye was removed by gel filtration. After blocking non-specific binding sites on the array, an incubation chamber was mounted onto the microarray to permit the loading of one control and one persistently *Mtb*-infected sample side-by-side on the same chip. Following sample incubation, unbound proteins were washed away. KAM-850 chips are spotted in duplicates with over 850 antibodies. 517 pan-specific antibodies used in the chip provide for the detection of 309 protein kinases, 38 protein phosphatases, 24 transcription factors, and 109 regulatory subunits of these enzymes and other cell signaling proteins. By this means, the microarrays provide information about the expression levels of these target proteins. 337 phosphosite-specific antibodies track the non-redundant phosphorylation of 157 sites in protein kinases, 6 sites in protein phosphatases, 51 sites in transcription factors, and 114 sites in other cell signaling proteins. By this means, the microarrays provide information about the activation state of the various kinases, which is usually linked to their phosphorylation state.

Each array produces a pair of 16-bit images captured with a Perkin-Elmer ScanArray Reader laser array scanner (Waltham, MA). Signal quantification was performed with ImaGene 8.0 from BioDiscovery (El Segundo, CA) with predetermined settings for spot segmentation and background correction. The background-corrected raw intensity data are logarithmically transformed with base 2. Since Z normalization in general displays greater stability as a result of examining where each signal falls in the overall distribution of values within a given sample, as opposed to adjusting all of the signals in a sample by a single common value, Z scores are calculated by subtracting the overall average intensity of all spots within a sample from the raw intensity for each spot, and dividing it by the standard deviations (SD) of all of the measured intensities within each sample [69]. Z ratios are further calculated by taking the difference between the averages of the observed protein Z scores and dividing by the SD of all of the differences for that particular comparison. Calculated Z ratios have the advantage that they can be used in multiple comparisons without further reference to the individual conditional standard deviations by which they were derived.

Specifically, persistently *Mtb*-infected THP-1 cells produced from four independent experiments along with non-infected controls were pooled to obtain one control sample and one persistently *Mtb*-infected sample. Based on our experience with similar experiments and studies performed by others [65, 70, 71], this approach reduces background signal noise resulting from experimental variations, but preserves signals underlying important changes.

### Data analysis

MetaCore software (Thomson Reuter) was used to generate shortest pathway interaction networks that are presented. Pathway specific GO filters or tissue specific filters were used to prioritize nodes and edges. MetaCore was further used to identify pathway associations of the identified input kinases.

## SUPPLEMENTARY MATERIAL FIGURES AND TABLE


