# Supplementary material for: Protein Phosphatase, Mg2+/Mn2+-dependent 1A controls the innate antiviral and antibacterial response of macrophages during HIV-1 and Mycobacterium tuberculosis infection
Source: Oncotarget. 2016 Mar 18;7(13):15394–409. doi: 10.18632/oncotarget.8190 (PMC4941249; doi:10.18632/oncotarget.8190)
Supplement: Supplementary file 1 [file oncotarget-07-15394-s001.pdf]

Protein Phosphatase, Mg^{2+}/Mn^{2+}-dependent 1A controls the innate antiviral and antibacterial response of macrophages during HIV-1 and *Mycobacterium tuberculosis* infection

Supplementary Material

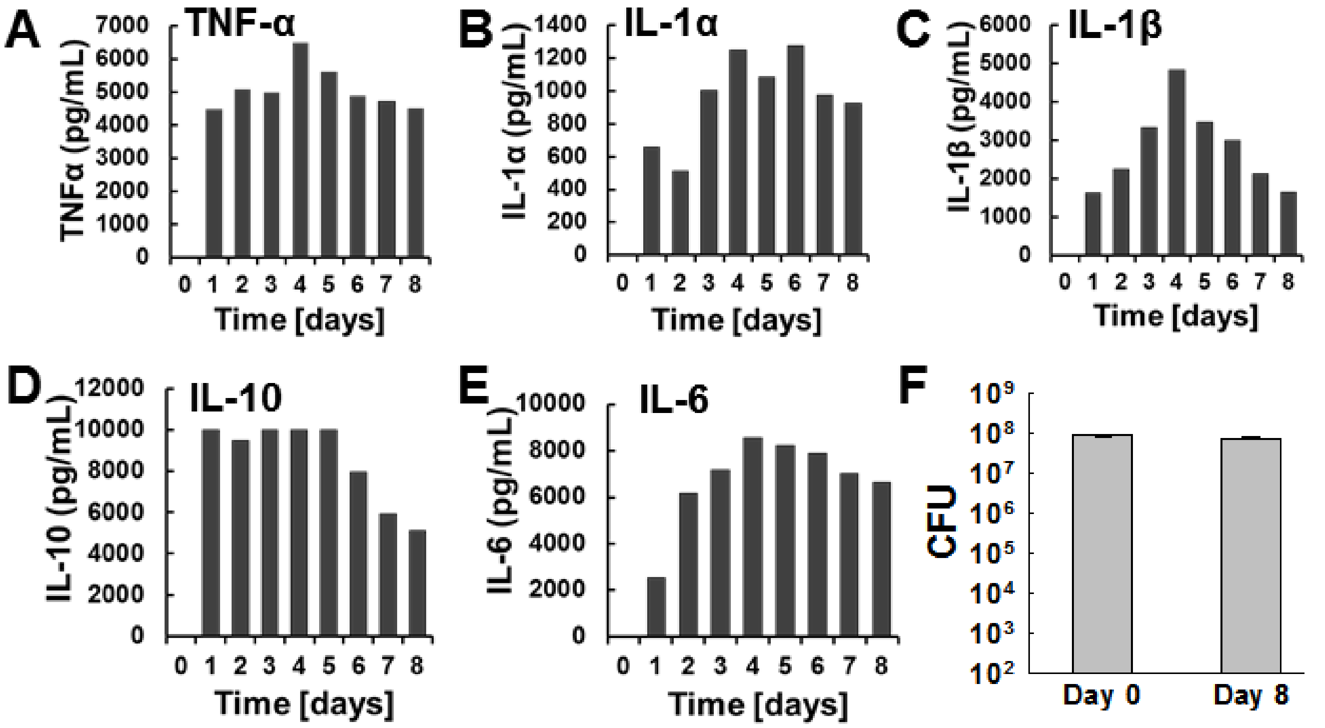

Supplementary Figure 1. Dynamic kinetic cytokine profiles produced by primary human monocytes during *Mtb*/macrophage aggregate formation. Culture supernatant from *Mtb*-infected (MOI 10) primary monocytes were collected and analyzed by Milliplex assays to quantify levels of (**A**) TNFα, (**B**) IL-1α, (**C)** IL-1β, (**D**) IL-10, and (**E**) IL-6. (**F**) *Mtb*-infected primary monocytes were harvested on day 8 and the number of viable bacteria was quantified relative to initial input on Day 0 by CFU plating. Data are expressed as the means ± standard deviations of three independent experiments.

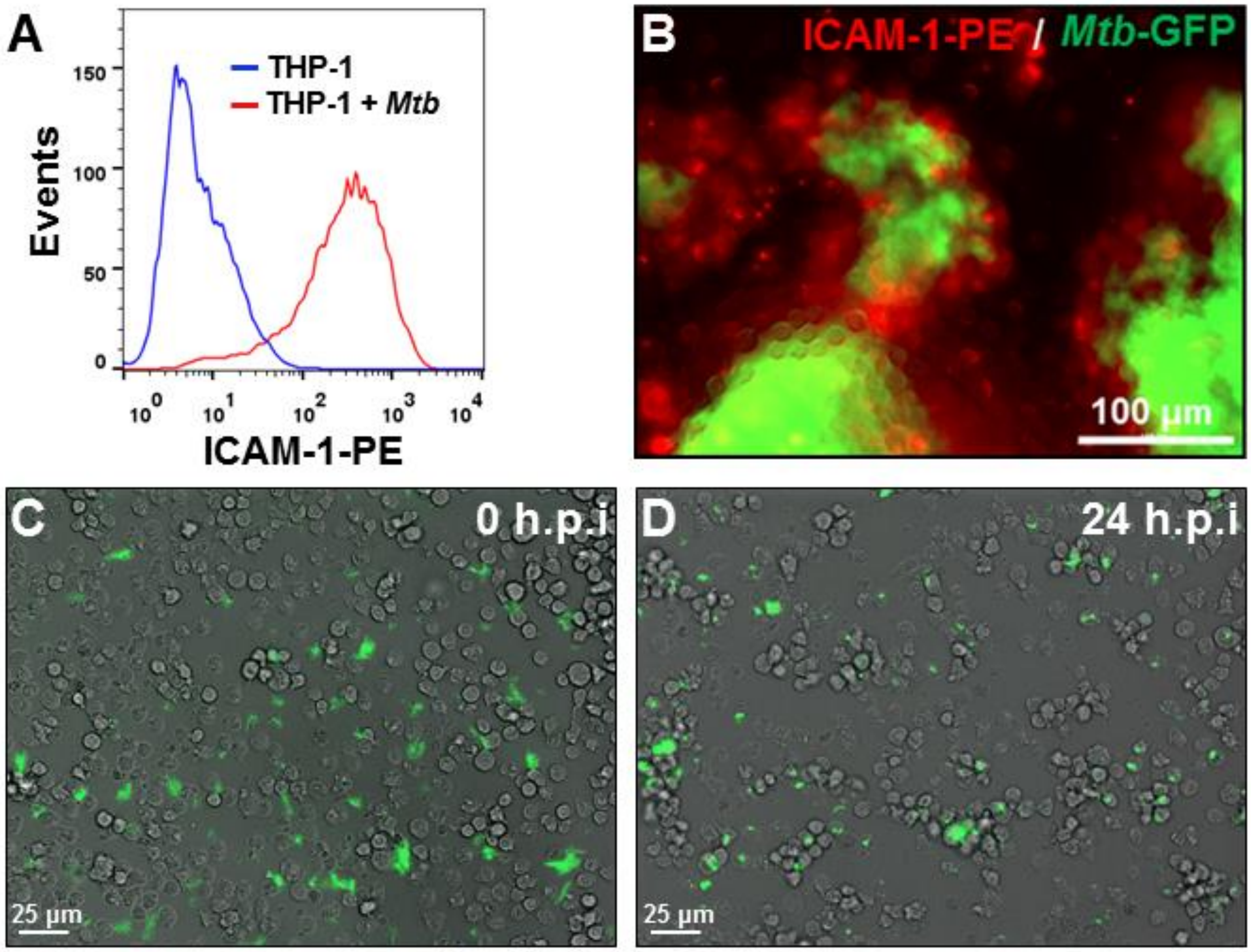

Supplementary Figure 2. Phagocytosis of *Mtb* and up-regulation of ICAM-1 in THP-1 monocytes. (**A**) THP-1 monocytes were infected with *Mtb*-GFP at an MOI of 25 and levels of ICAM-1 48 h.p.i were quantified by surface staining with antibody to ICAM-1 using flow cytometry. (**B**) *Mtb*/macrophage aggregates were stained with ICAM-1-PE (red) antibody 2 weeks post infection to visualize up-regulation of ICAM-1 after *Mtb* (green) infection. THP-1 monocytes were infected with *Mtb*-GFP at an MOI of 25 and phagocytosis was followed by fluorescence microscopy. Representative images of merged bright field and GFP channels are shown (**C**) at 0 h.p.i and (**D**) 24 h.p.i.

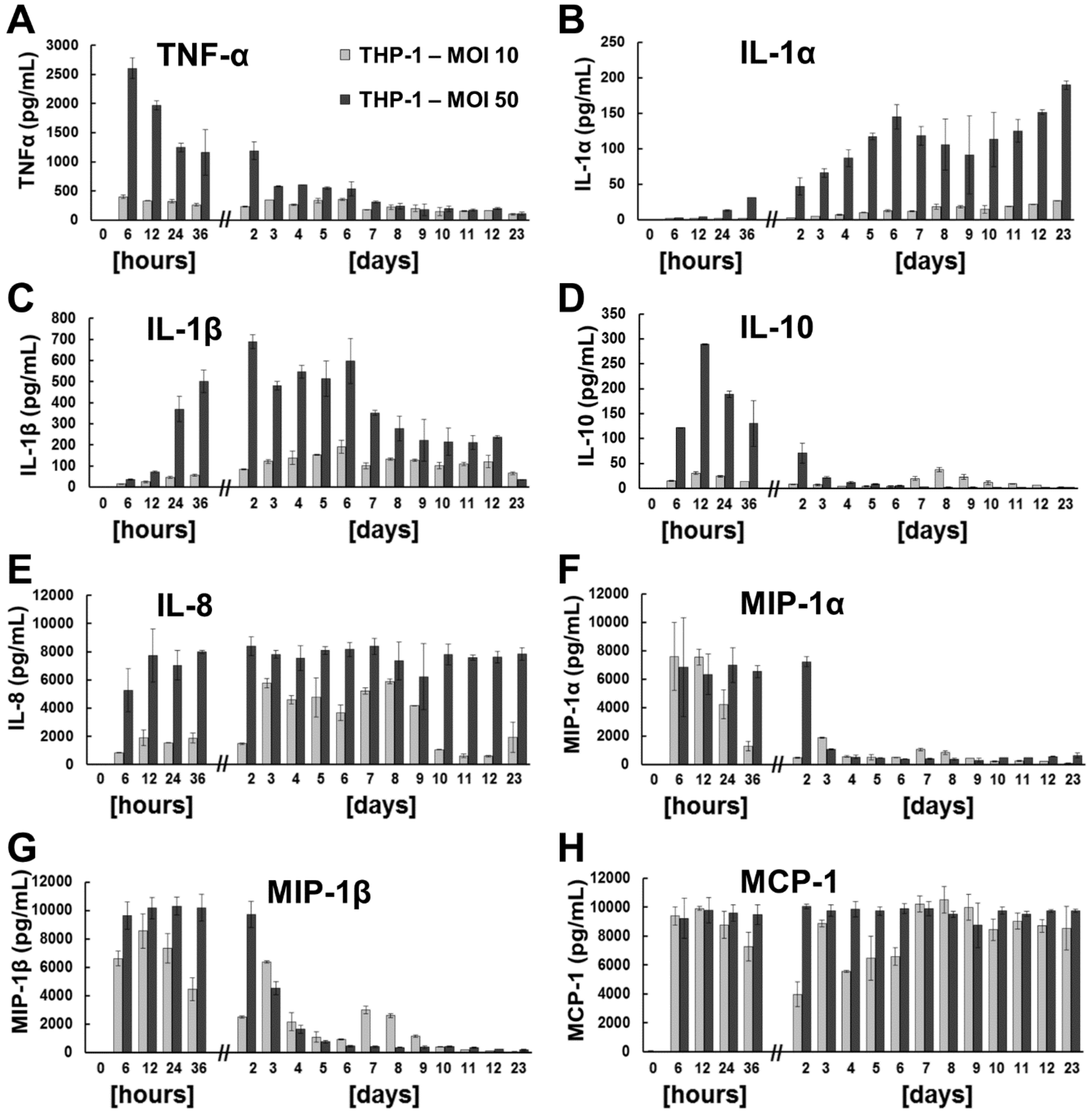

Supplementary Figure 3. Dynamic kinetic cytokine and chemokine profiles produced during THP-1 cell aggregation in response to *Mtb*-infection. Culture supernatant from *Mtb*-infected (MOI 10 or 50) THP-1 cells were collected and analyzed by Milliplex assays to quantify levels of (**A**) TNFα, (**B**) IL-1α, (**C**) IL-1β, (**D**) IL-10, (**E**) IL-8, (**F**) MIP-1α, (**G**) MIP-1β, and (**H**) MCP-1. Data are expressed as the means ± standard deviations of three independent experiments.

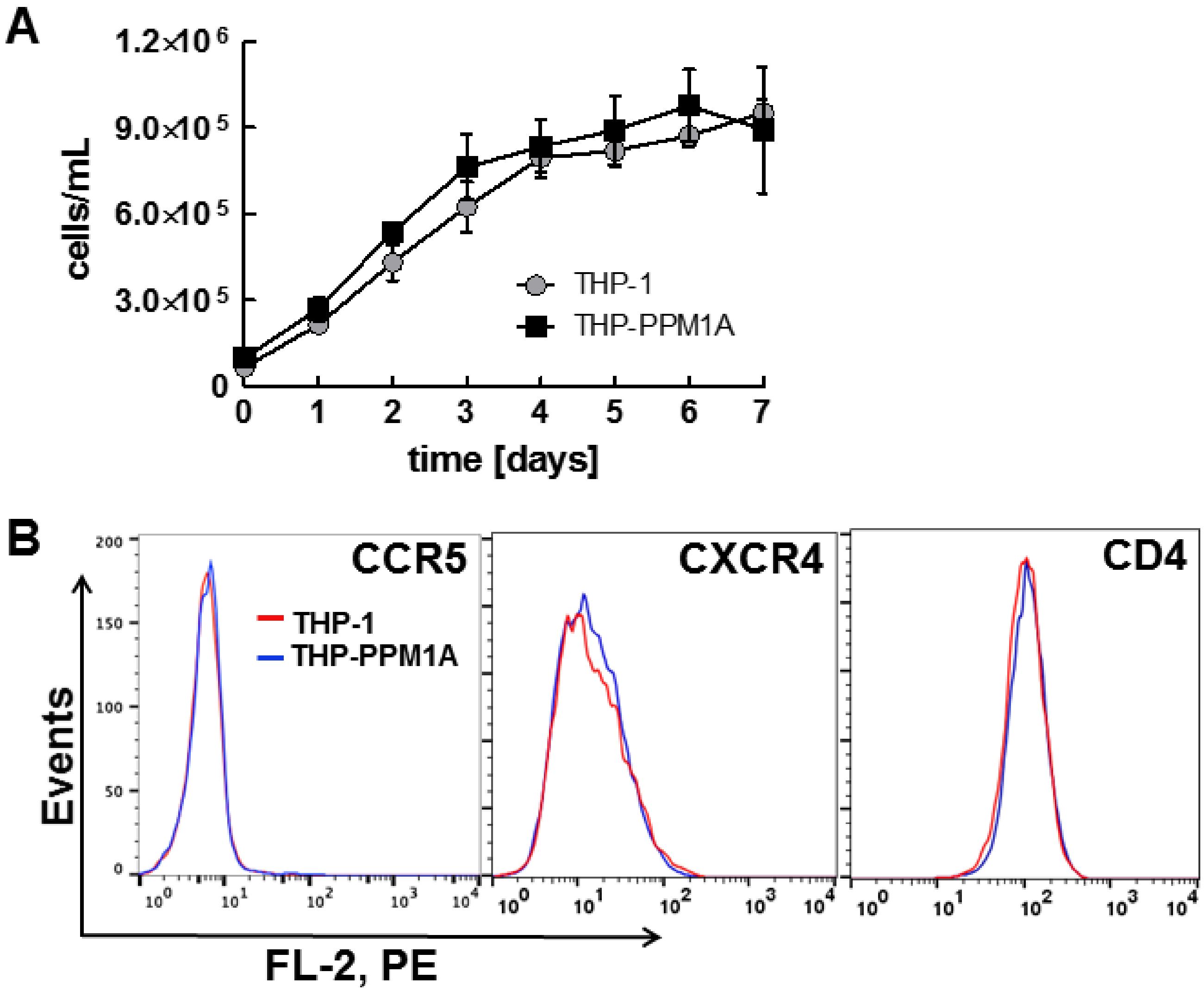

Supplementary Figure 4. THP-PPM1A cells exhibit no marked differences in proliferation or expression of HIV-1 receptors. (**A**) Proliferation of THP-1 or THP-PPM1A cells were monitored by daily cell counts using flow cytometry for a period of one week. (**B**) THP-1 or THP-PPM1A cells were stained with PE-conjugated antibodies to CD4, CD184 (CXCR4), or CD195 (CCR5) and analyzed by flow cytometry. FL-2 signal indicates the presence and amount of cell surface markers.

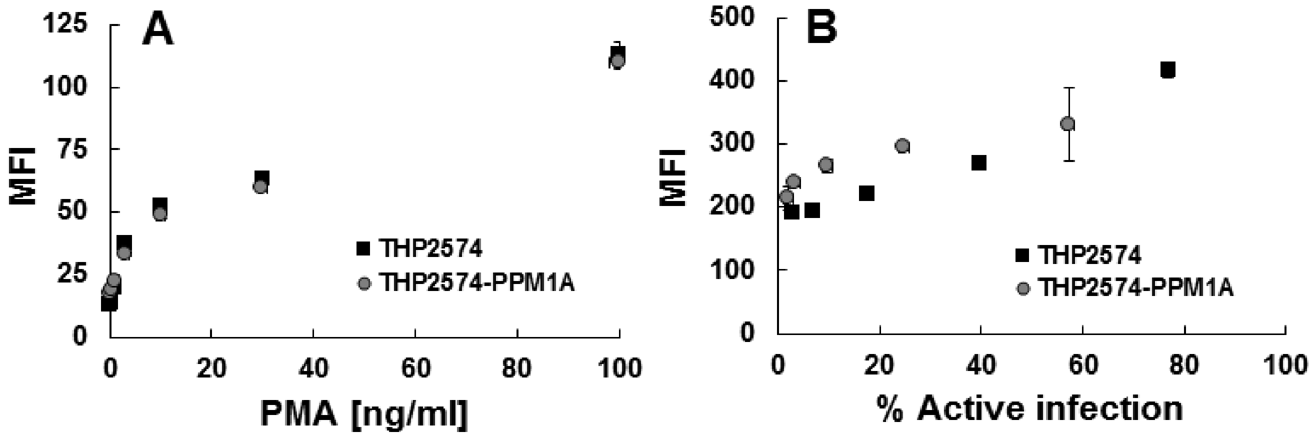

Supplementary Figure 5. PPM1A expression levels have no effect on Tat-dependent or independent HIV-1 LTR activity. (**A**) THP2574 and THP2574-PPM1A cells were stimulated with the PKC/NF-κB activating phorbol ester PMA and measured for LTR-induction by using GFP expression as a surrogate marker. (**B**) THP2574 and THP2574-PPM1A cells were retrovirally transduced with a MSCV-Tat expression vector, and levels of Tat-induced, LTR-driven GFP expression was analyzed by flow cytometry normalized to active infection levels. Data in this figure represent the means ± standard deviations of three independent experiments.

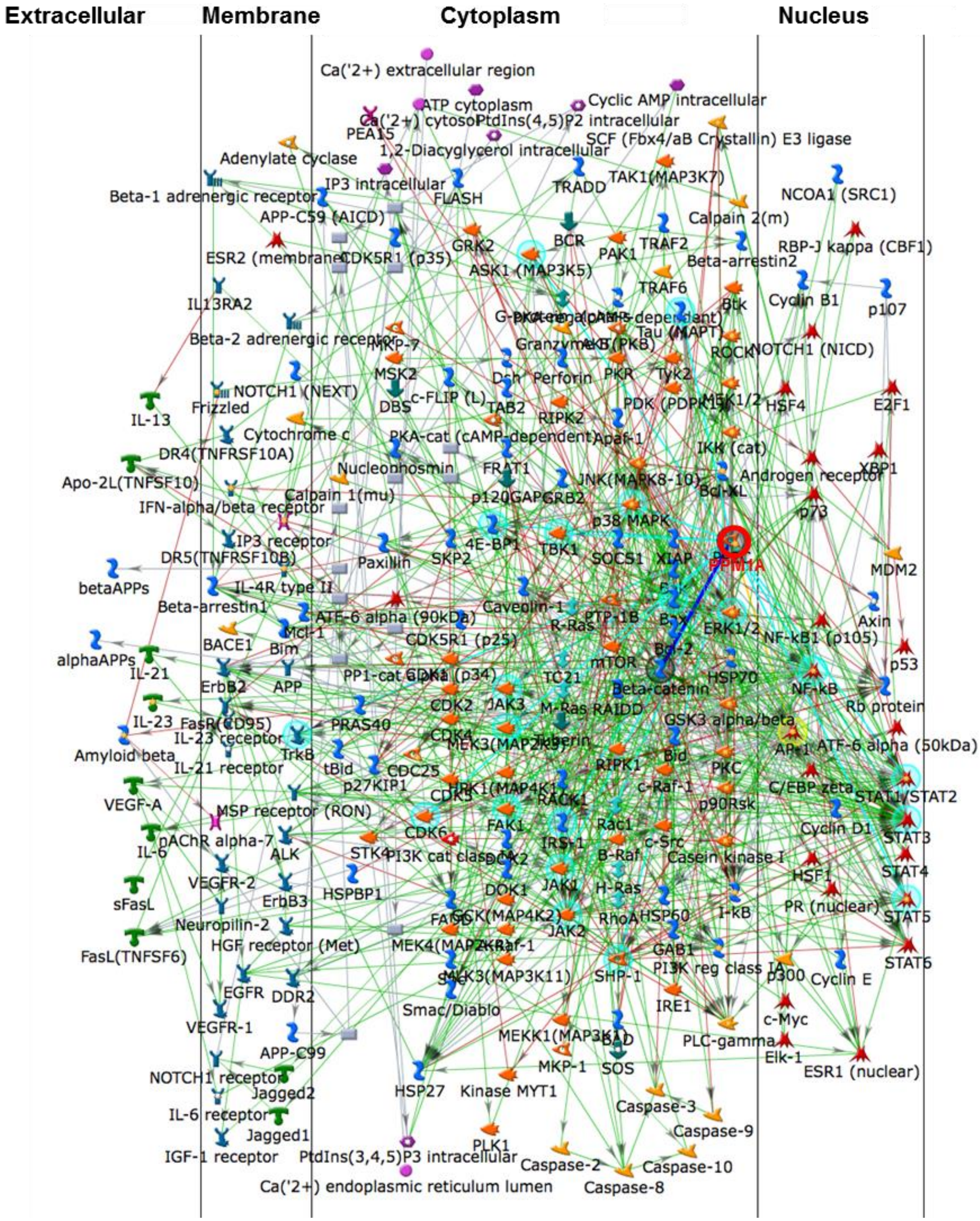

Supplementary Figure 6. Predicted protein-protein interaction network of persistently *Mtb* infected macrophages. MetaCore analysis showing a one-step shortest pathway protein-protein interaction network using the proteins that were found altered in persistently Mtb infected macrophages as seed nodes to predict possible up- and down-stream effects of the observed changes in the kinome profile of persistently *Mtb* infected macrophages. The PPM1A seed node is marked by a red circle and the directly connected edges are indicated by blue lines. Directly connected proteins are indicated by blue circles.

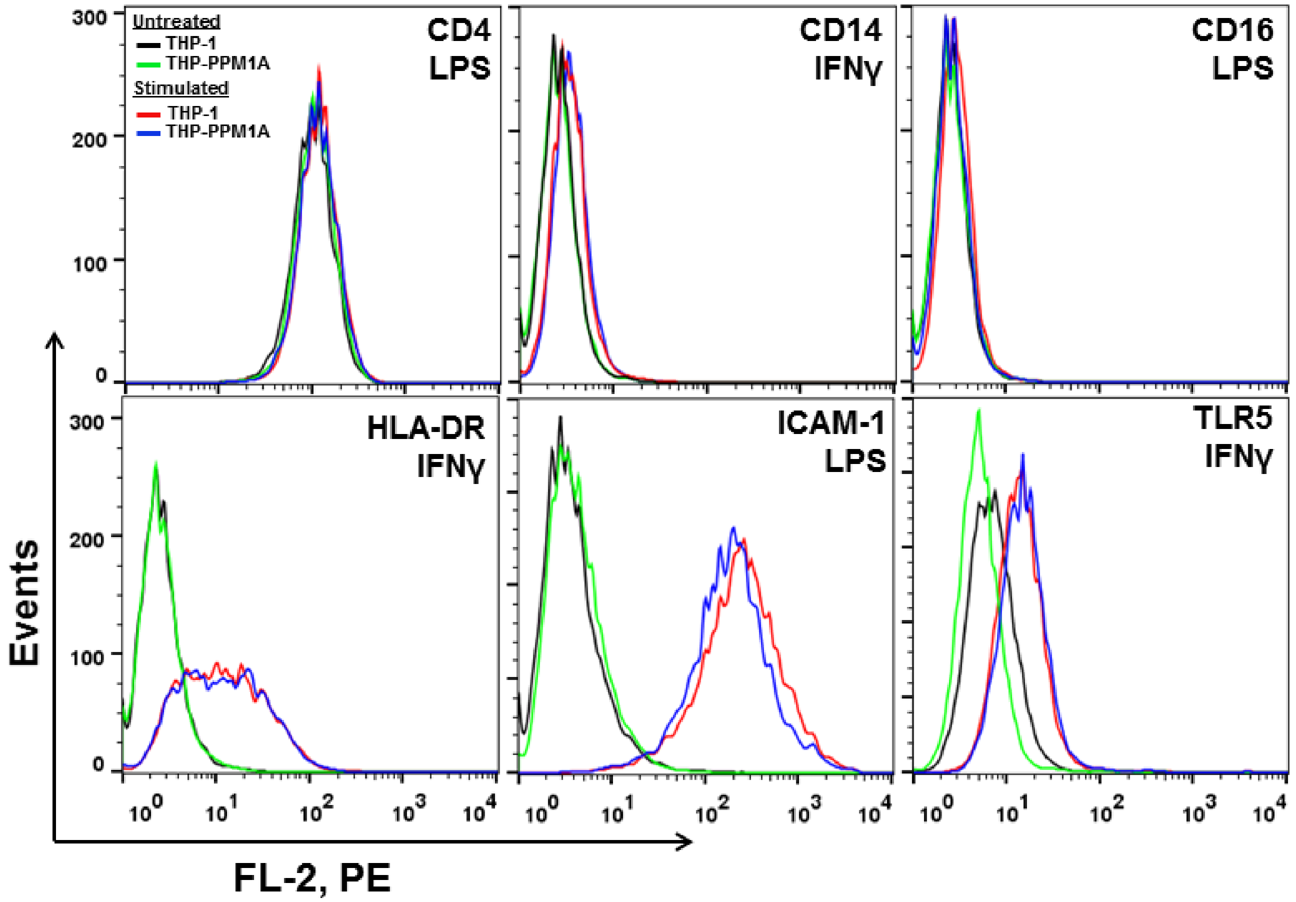

Supplementary Figure 7. THP-PPM1A cells exhibit no marked differences in cell surface marker expression in response to stimuli compared to the parent THP-1 strain. THP-1 or THP-PPM1A cells were unstimulated or stimulated with LPS/IFNɣ for 24 h. Cells were then stained with PE-conjugated antibodies to CD4, CD14, CD16, HLA-DR, ICAM-1, or TLR5 and analyzed by flow cytometry. FL-2 signal indicates the presence and amount of cell surface marker.

SUPPLEMENTARY TABLES

Target Protein Name	Phospho Site (Human)	Full Target Protein Name	Z-ratio (THP-1 *Mtb*/ THP-1)
STAT2	Pan-specific	Signal transducer and activator of transcription 2	**-5.84**
Catenin b1	Pan-specific	Catenin (cadherin-associated protein) beta 1	**-5.61**
ErbB2 (HER2)	Pan-specific	ErbB2 (Neu) receptor-tyrosine kinase	**-4.45**
Bax	Pan-specific	Apoptosis regulator Bcl2-associated X protein	**-4.43**
Trail	Pan-specific	Tumor necrosis factor-related apoptosis-inducing ligand	**-4.08**
IkBb	Pan-specific	Inhibitor of NF-kappa-B beta (thyroid receptor interacting protein 9)	**-3.85**
STAT4	Pan-specific	Signal transducer and activator of transcription 4 (acute phase response factor)	**-3.81**
Fos	Pan-specific	Fos-c FBJ murine osteosarcoma oncoprotein-related transcription factor	**-3.77**
IkBa	Pan-specific	Inhibitor of NF-kappa-B alpha (MAD3)	**-3.72**
STAT1a	Pan-specific	Signal transducer and activator of transcription 1 alpha	**-3.60**
KAP	Pan-specific	Cyclin-dependent kinase associated phosphatase (CDK inhibitor 3, CIP2)	**-3.56**
STAT3	Pan-specific	Signal transducer and activator of transcription 3 (acute phase response factor)	**-3.21**
STAT5A	Pan-specific	Signal transducer and activator of transcription 5A	**-3.18**
STAT6	Pan-specific	Signal transducer and activator of transcription 6	**-3.16**
STAT5B	Pan-specific	Signal transducer and activator of transcription 5B	**-2.75**
NFkappaB p65	Pan-specific	NF-kappa-B p65 nuclear transcription factor	**-2.64**
Rac1	Pan-specific	Ras-related C3 botulinum toxin substrate 1	**-2.45**
Hsp27	S82	Heat shock 27 kDa protein beta 1 (HspB1)	**-2.30**
NFkappaB p50	Pan-specific	NF-kappa-B p50 nuclear transcription factor	**-2.10**
APG2	Pan-specific	Hsp 70-related heat shock protein 4 (HSP70RY)	**-2.08**
Paxillin 1	Pan-specific	Paxillin 1	**-2.07**
PI3K p85/p55	Pan-specific	Phosphatidylinositol 3-kinase regulatory subunit alpha	**-2.01**
GSK3a	Pan-specific	Glycogen synthase-serine kinase 3 alpha	**-1.92**
Bcr	Y177	Breakpoint cluster region protein	**-1.81**
GRK2 (BARK1)	Pan-specific	G protein-coupled receptor-serine kinase 2	**-1.76**
ASK1 (MAP3K5)	S966	Apoptosis signal regulating protein-serine kinase	**-1.76**
Bcl-xS/L	Pan-specific	Bcl2-like protein 1	**-1.75**
B23 (NPM)	T199	B23 (nucleophosmin, numatrin, nucleolar protein NO38)	**-1.71**
RONa	Pan-specific	Macrophage-stimulating protein receptor alpha chain	**-1.69**
ASK1 (MAP3K5)	Pan-specific	Apoptosis signal regulating protein-serine kinase	**-1.67**
TRADD	Pan-specific	Tumor necrosis factor receptor type 1 associated DEATH domain protein	**-1.66**
Rb	Pan-specific	Retinoblastoma-associated protein 1	**-1.58**
GCK	Pan-specific	Germinal centre protein-serine kinase	**-1.57**
Hpk1 (MAP4K1)	Pan-specific	Hematopoetic progenitor protein-serine kinase 1	**-1.56**
HSF4	Pan-specific	Heat shock transcription factor 4	**-1.49**
RIP2/RICK	Pan-specific	Receptor-interacting serine/threonine-protein kinase 2 (RIPK2)	**-1.42**
FasL	Pan-specific	Tumor necrosis factor ligand, member 6	**-1.39**
ErbB3	Y1328	Tyrosine kinase-type cell surface receptor HER3	**-1.39**
Grp78	Pan-specific	Glucose regulated protein 78	**-1.38**
APP	T668	Amyloid beta A4 protein	**-1.33**
Arrestin b1	Pan-specific	Arrestin beta 1	**-1.32**
p107	Pan-specific	Retinoblastoma (Rb) protein-related p107 (PRB1)	**-1.32**
hHR23B	Pan-specific	UV excison repair protein RAD23 homolog B	**-1.29**
CK1d	Pan-specific	Casein protein-serine kinase 1 delta	**-1.24**
PLCg1	Y783	1-phosphatidylinositol-4,5-bisphosphate phosphodiesterase gamma-1	**-1.23**
MST1	Pan-specific	Mammalian STE20-like protein-serine kinase 1 (KRS2)	**-1.20**
MST3	Pan-specific	Mammalian STE20-like protein-serine kinase 3	**-1.20**
NFkappaB p65	S529	NF-kappa-B p65 nuclear transcription factor	**1.20**
CDK6	Pan-specific	Cyclin-dependent protein-serine kinase 6	**1.21**
FAK	Y397	Focal adhesion protein-tyrosine kinase	**1.21**
PKCg	T514	Protein-serine kinase C gamma	**1.23**
PKCg	T655	Protein-serine kinase C gamma	**1.25**
JAK1	Y1034	Janus protein-tyrosine kinase 1	**1.26**
JAK1	Pan-specific	Janus protein-tyrosine kinase 1	**1.31**

JNK1/2/3	Pan-specific	Jun N-terminus protein-serine kinase (stress-activated protein kinase (SAPK)) 1/2/3	**1.32**
Erk1 (MAPK3)+ Erk2 (MAPK1)	Y204	Extracellular regulated protein-serine kinase 1 (p44 MAP kinase)+Extracellular regulated protein-serine kinase 2 (p42 MAP kinase)	**1.34**
Erk1 (MAPK3)+ Erk2 (MAPK1)	T202	Extracellular regulated protein-serine kinase 1 (p44 MAP kinase)+Extracellular regulated protein-serine kinase 2 (p42 MAP kinase)	**1.35**
Tau	S717	Microtubule-associated protein tau	**1.37**
PKCa	Pan-specific	Protein-serine kinase C alpha	**1.45**
Cyclin B1	Pan-specific	Cyclin B1	**1.45**
PKCd	Y313	Protein-serine kinase C delta	**1.49**
Hsp60	Pan-specific	Heat shock 60 kDa protein 1 (chaperonin, CPN60)	**1.53**
pThr(MmAb)	pThr	pThr(MmAb)	**1.56**
FAS	Pan-specific	Tumor necrosis factor superfamily member 6 (Apo1, CD95)	**1.60**
PKCb1	Pan-specific	Protein-serine kinase C beta 1	**1.62**
Crystallin aB	Pan-specific	Crystallin alpha B (heat-shock 20 kDa like-protein)	**1.66**
MEK3 (MAP2K3)	Pan-specific	MAPK/ERK protein-serine kinase 3 (MKK3)	**1.68**
4E-BP1	Pan-specific	Eukaryotic translation initiation factor 4E binding protein 1 (PHAS1)	**1.70**
p73	Pan-specific	Tumor suppressor protein p73	**1.74**
CK1e	Pan-specific	Casein protein-serine kinase 1 epsilon	**1.78**
PKCd	Y313	Protein-serine kinase C delta	**1.79**
FAK	Pan-specific	Focal adhesion protein-tyrosine kinase	**2.16**
Cdc25B	Pan-specific	Cell division cycle 25B phosphatase	**2.73**
PPM1A	Pan-specific	Protein-serine phosphatase 2C - catalytic subunit - alpha	**8.59**

Supplementary Table 1. Kinome array analysis of persistently *Mtb*-infected THP-1 cells
